# A “Kissing-Stents” Technique in the Management of Afferent Limb Syndrome With Concomitant Efferent Limb Obstruction in a Patient With Gastric Cancer and Billroth II Anatomy

**DOI:** 10.14309/crj.0000000000000266

**Published:** 2020-01-07

**Authors:** Sami Elamin, Daniel Stein, Jonah Cohen

**Affiliations:** 1 Department of General Internal Medicine, Massachusetts General Hospital, Boston, MA; 2 Department of Medicine, Harvard Medical School, Boston, MA; 3 Department of Gastroenterology, Beth Israel Deaconess Medical Center, Boston, MA; 4 Center for Advanced Endoscopy, Beth Israel Deaconess Medical Center, Boston, MA

## Abstract

The “kissing-stents” technique has been used in endovascular interventions for the management of aortic and arterial stenosis at bifurcation sites. However, to our knowledge, the use of this technique to prevent stent migration in endoscopy has not been described to date. We present a 65-year-old man with metastatic gastric adenocarcinoma status post-Billroth II gastrojejunostomy complicated by simultaneous afferent and efferent limb syndrome with gastric outlet obstruction and biliary dilatation. Two uncovered metal stents were used to relieve the afferent and efferent loop obstructions. These 2 stents were anchored together in a “kissing-stents” technique and using a clip to prevent migration. The patient was able to resume oral intake, and his liver function tests improved. This intervention should be considered in other cases of advanced malignancies causing obstructions for curative or palliative intent.

## INTRODUCTION

Afferent limb (loop) syndrome is a known complication in patients who undergo partial gastrectomy with Billroth II reconstruction. Obstruction of the afferent (biliary) limb results in increased intraluminal pressure and distention from the accumulation of enteric secretions and bile. Back pressure is transmitted to the biliopancreatic ductal system, which can lead to ascending cholangitis and pancreatitis. Similarly, obstruction of the efferent limb, as seen, for example, in patients with malignancy, causes functional small bowel or gastric outlet obstruction. Patients classically present with nausea, emesis, and abdominal pain. In severe cases of afferent loop syndrome, high luminal pressures and distention increase bowel wall tension in the afferent loop and can lead to ischemia and gangrene with subsequent perforation and peritonitis. We present a case of concurrent afferent and efferent limb obstruction secondary to a gastric mass in the setting of remote Billroth II gastrojejunostomy for peptic ulcer disease, treated with metal stents deployed into the afferent and efferent limbs. To prevent stent migration, a “kissing-stents” technique was used, and 2 clips were deployed to attach the gastric end of both metal stents.

## CASE REPORT

A 65-year-old man with a history of peptic ulcer disease status postremote Billroth II gastrojejunostomy in 1970 with known metastatic gastric carcinoma presented in the outpatient setting with several months of abdominal pain, fatigue, weight loss, and poor appetite. His body mass index was 15 kg/m^2^. This constellation of symptoms resulted in interruption of his chemotherapy regimen. On examination, his abdomen was soft and initially nondistended. He had hyperactive bowel sounds, normal pitch. There was moderate tenderness to palpation over the right upper quadrant and left side, no rebound, and no guarding. The laboratory results revealed anemia hemoglobin 7.7 g/dL, alanine aminotransferase 43 U/L, aspartate aminotransferase 45 U/L, alkaline phosphatase 2006 U/L, TBili 0.5 mg/dL, and albumin 2.5 g/dL. He was admitted to the hospital for management.

**Figure 1.**
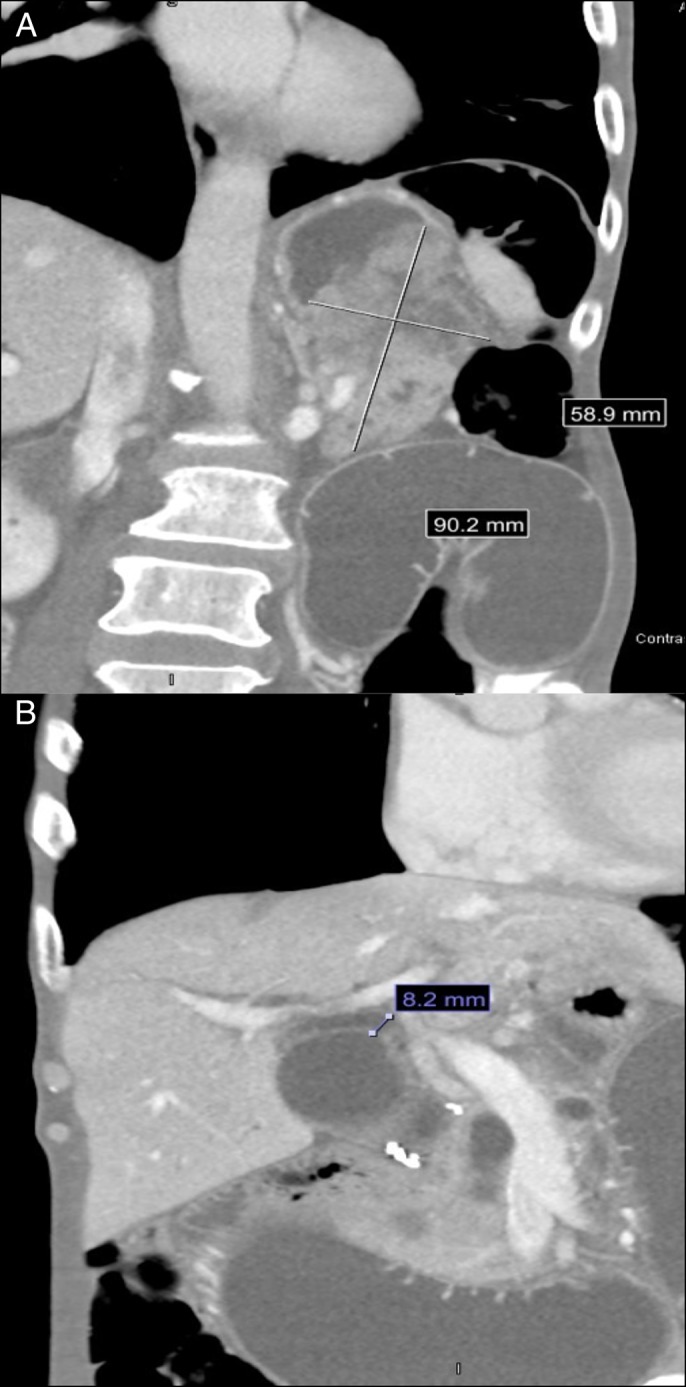
(A) High-grade afferent loop obstruction due to enlarging gastric mass with probable extragastric extension into the left upper quadrant and decompressed distal bowel. (B) Biliary and ductal dilatation, gallbladder dilatation, and pancreatic ductal dilatation.

Abdominal computed tomography showed Billroth II gastrojejunostomy with high-grade afferent loop obstruction due to enlarging gastric mass with probable extragastric extension into the left upper quadrant and decompressed distal bowel. There was contact without definite invasion of the pancreatic tail and lateral limb of the left adrenal gland. Interval increase in biliary ductal dilatation, worst in the left hepatic lobe with concomitant gallbladder, and pancreatic duct distension were noted all consistent with downstream obstruction (Figure 1). Given his body mass index of 15 and notable deconditioning, he was deemed a priori not to be a surgical candidate for a palliative bypass.

**Figure 2.**
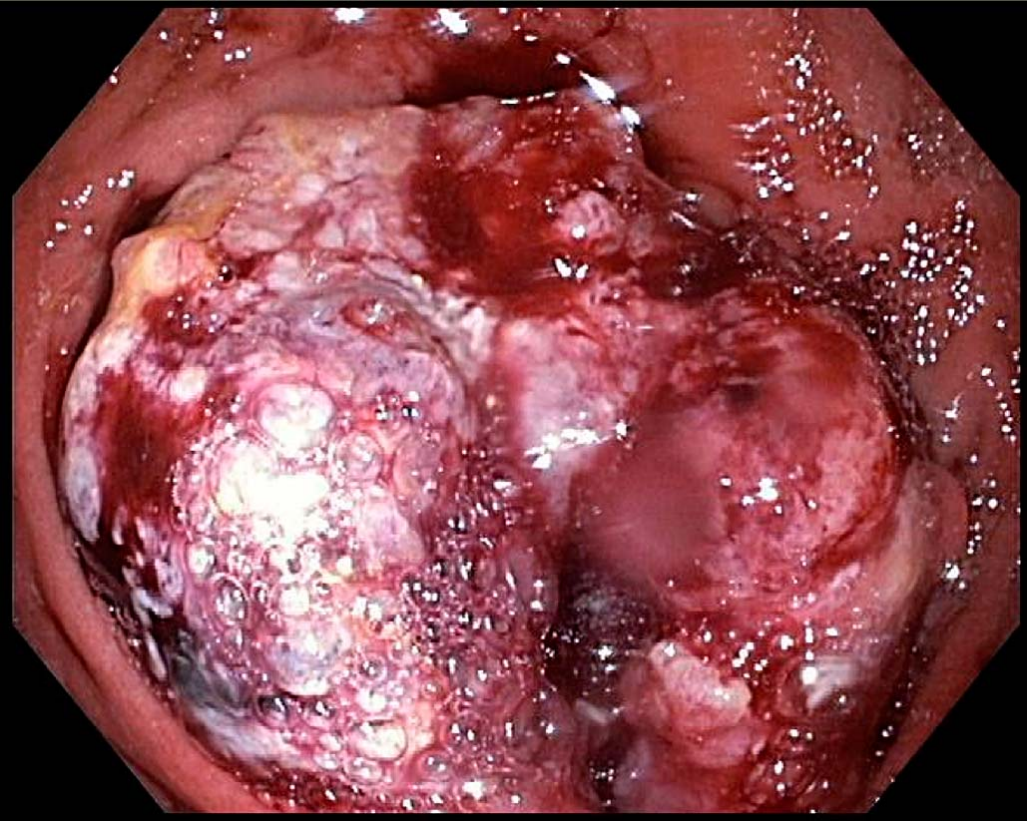
Gastric mass.

**Figure 3.**
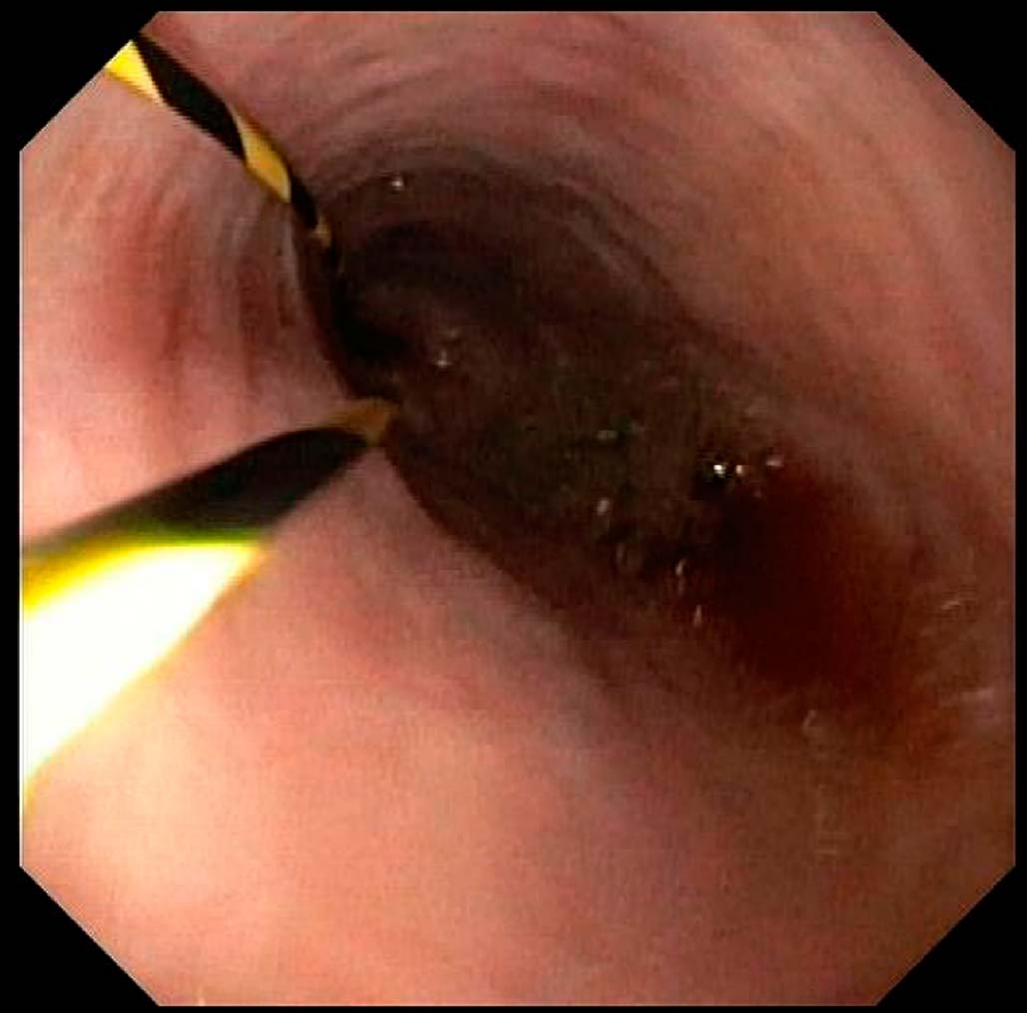
Double-guidewire technique.

**Figure 4.**
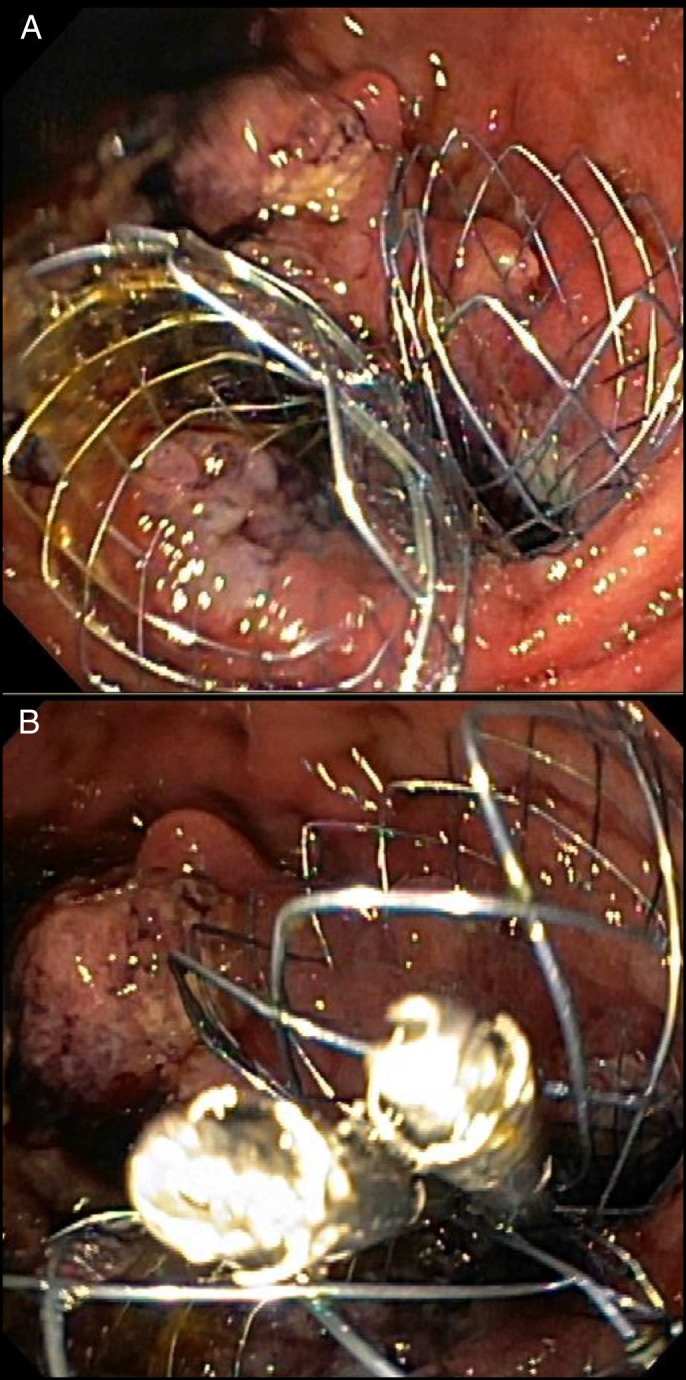
(A) Afferent and efferent limb stents. (B) Afferent and efferent limb stents anchored together with a clip to prevent migration, demonstrating a “kissing-stents” technique.

**Figure 5:**
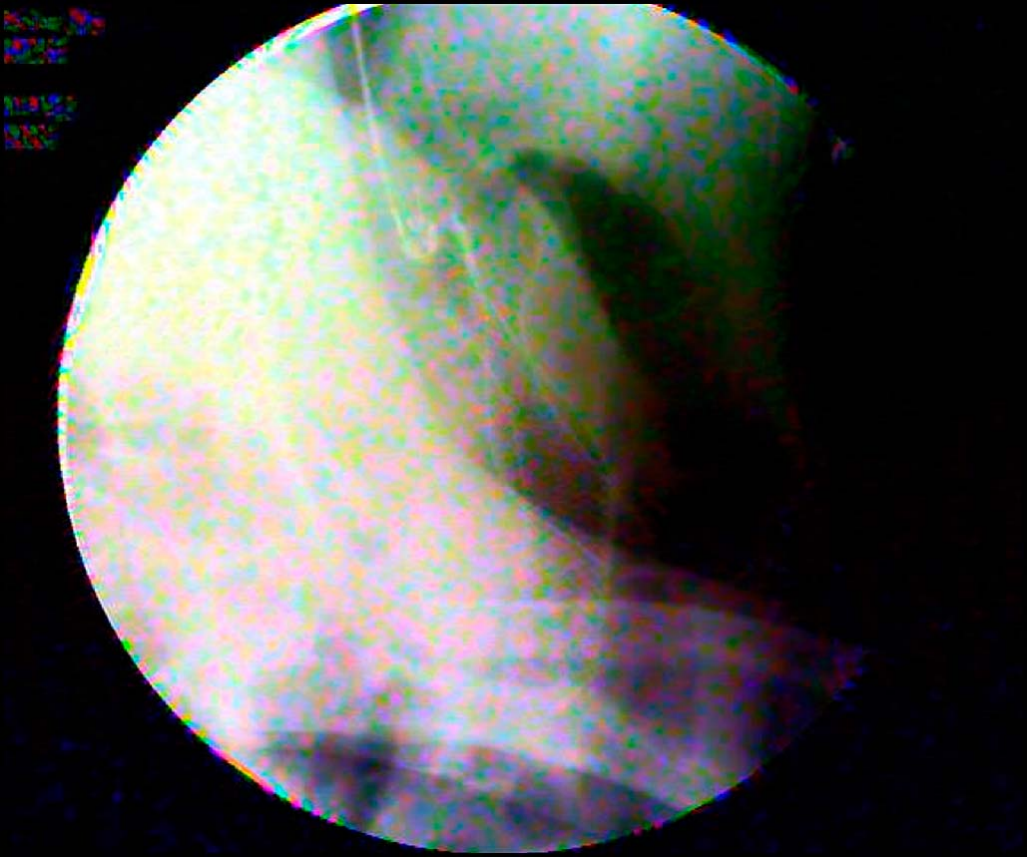
Fluoroscopy showing afferent and efferent limb stents anchored with a clip.

Upper endoscopy showed a circumferential, fungating, and ulcerated friable 4–5 cm mass of malignant appearance involving the anastomosis. The mass caused near-total obstruction of both the pancreatobiliary and efferent limbs (Figure 2). Because of the severity of his obstruction, a pediatric gastroscope was required to pass the tumor. A guidewire was passed into the afferent limb through the pediatric gastroscope after which a second guidewire was passed into the efferent limb (Figure 3). The guidewires were both passed before any stent deployment, given a concern for possible worsening obstruction after the first stent was placed and expanded. The afferent limb guidewire was backloaded through a duodenal stent loaded in a therapeutic gastroscope, and the gastroscope was advanced through the wire. An uncovered metal stent was deployed across the gastric mass into the afferent limb under endoscopic and fluoroscopic guidance. The efferent limb guidewire was then backloaded through a second duodenal stent loaded in the therapeutic gastroscope, and the gastroscope was advanced over the wire. A second uncovered metal stent was deployed across the gastric mass into the efferent limb under endoscopic and fluoroscopic guidance (Figure 4). The afferent limb was deployed first because there was concern that the first stent would obstruct the lumen and ability to place the second stent. In addition, afferent limb obstruction was the most acute pathology, as evident by this patient's presenting symptoms, laboratory tests, and imaging. Using the double guidewire technique was critical in ensuring our ability to properly deploy the second stent. Without a double wire technique, given the degree of obstruction, it would likely not have been possible to introduce a wire down the efferent limb after afferent limb stent deployment. To prevent stent migration, a “kissing-stents” technique was used, and 2 clips were deployed to attach the gastric end of both metal stents (Figure 5).

The patient returned to the floor. After the procedure, he was able to resume a clear liquid diet the first day, followed by a duodenal stent diet. Liver function tests normalized except for alkaline phosphatase, which continued to downtrend slowly (

**Table 1.**
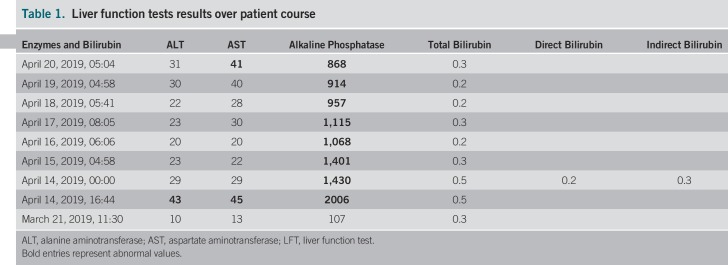
Liver function tests results over patient course

Table 1). Stents remain patent because of the time of this report, and he was tolerating oral intake.

## DISCUSSION

There are currently few studies on the use of metallic stents in the obstruction at the bifurcation of a surgically reconstructed intestine, and the safety and efficacy of the procedure have not been elucidated.^1^ Placement of afferent and efferent limb stents can allow resumption of oral intake in patients with afferent limb syndrome with efferent limb obstruction. This is an uncommon complication of Billroth II reconstruction but could likely be applied to another postsurgical anatomy such as hepaticojejunostomy for pancreatic resection. Anchoring of the 2 stents with a clip, using a “kissing-stents” technique, is a novel way to prevent stent migration.^1–3^ Another method to prevent stent migration is suturing the stents to the gastric wall. We elected to clip the 2 stents together because we felt confident this would successfully prevent migration by forming kissing stents. Given the size and shape of the patient's stomach, suturing to an adjacent wall would have been difficult and unlikely to be more successful than the endoclip approach we created. A study comparing the outcomes of those 2 techniques would be useful.^1^ Consideration should always be given to a double-wire technique to prevent an initial stent from occluding the second lumen and preventing passage. Despite the typically advanced malignancies causing these obstructions, intervention should be offered if it would provide therapeutic or palliative benefit.

## DISCLOSURES

Author contributions: S. Elamin wrote the manuscript, reviewed the literature, and is the article guarantor. D. Stein reviewed the literature and revised the manuscript. J. Cohen approved the final version.

Financial disclosure: None to report.

Informed consent was obtained for this case report.
